# Gynecology and Obstetrics

**Published:** 1894-02

**Authors:** 


### GYNECOLOGY AND OBSTETRICS.

EDITED BY DR. VIRGIL O. HARDON.

### THE TREATMENT OF UTERINE FIBROIDS.

Dr. H. T. Byford, of Chicago, in the *American Gynecological Journal*, thus speaks of the treatment of uterine fibroids: There are certain facts in connection with the growth of uterine fibroids which we may set down as true, or approximately true, upon which we may base principles of treatment.

*First.*—Interstitial uterine fibroids are of slow growth, and usually stop growing at the menopause.

*Second.*—Extrauterine pedunculated and intrauterine polypoid fibroids, and those that project from the uterine walls (subserous and submucous), show less tendency to stop growing than the interstitial variety.

*Third.*—A multitude of interstitial tumors causes a rapid increase in the size of the uterus, but after a time outgrow and, by crowding together, interfere with their nutrient supply, stop growing, and undergo degenerative changes.

*Fourth.*—Single tumors show but little tendency to stop growing, and, when interfered with in their development, are apt to undergo œdematous or cystic degenerative changes. The same may be said if two or three nodules develop as an isolated mass.

*Fifth.*—Cystic fibroids grow in spite of treatment or the supervention of the menopause.

*Sixth.*—Uterine polypi are curable only by removal.

In treating these tumors we must select our remedies according to the situation and relations of the tumors. There are six different methods of treatment to be considered.

*First.*—The alterative treatment, which has no effect whatever, and is only successful in those cases that get well without any treatment. It is utterly irrational, and injurious to the health of the patient.

*Second.*—The ergot treatment causes expulsion of the polypi. It diminishes the vascularity of the uterine walls, and thus checks the growth of interstitial tumors, and retards the growth of the sub-peritoneal. By the additional influence of pressure upon the submucous fibroids it causes their atrophy, or intrusion into the uterine cavity as polypi. It hastens the spontaneous atrophy of the multiple fibromata. It also acts symptomatically in checking the hemorrhage, and often enables the patient to wait for the menopause. It is of great value near the menopause and is a safe method.

*Third.*—Dilatation, curetting and treatment of the endometrium may relieve the endometritis and thus check hemorrhage and diminish the tendency to rapid growth.

*Fourth.*—Electricity in large dosage stimulates the uterus to contraction and thus acts slightly like ergot. The positive intrauterine electrode cicatrizes and contracts the uterine cavity, relieving hemorrhage and compressing submucous fibroids. Its action does not extend far from the electrode, as is proved by the fact (demonstrated by Dr. F. H. Martin) that all parts of the uterine cavity contiguous to the tumor must be touched. The negative intrauterine pole liquefies and destroys superficial layers of the tumor, and, in connection with subsequent application of the positive pole, interferes with their nutrition. Electricity is applicable to interstitial submucous fibroids that do not project much into the uterine cavity. Subserous and multiple growths and hard tumors, wherever situated, are not much affected. In properly selected cases the method is quite a safe one, although the mortality would be quite large if employed by inexperienced or careless operators.

*Fifth.*—Removal of the uterine appendages temporarily diminishes the vascularity of the uterus, but acts chiefly by inaugurating an artificial menopause. It can be depended upon in small tumors situated well above the internal os. Tumors larger than a cocoanut, particularly those that have contracted adhesions, are but little affected by it. The operation is quite safe for small tumors, but is often difficult and dangerous when the parts are displaced and diseased by larger ones.

*Sixth.*—Hysterectomy is, of course, a radical cure. Vaginal hysterectomy, for small tumors, is quite safe, but is seldom indicated for small tumors unless accompanied by excessive incurable hemorrhage or pain. Abdominal hysterectomy is becoming daily less formidable, partly because of improved methods and technique, and partly because the tumors are, at the present day, not often allowed to become so large as to contract adhesions, or to lift up the broad ligaments and become involved too extensively with the pelvic organs and tissues.

With regard to the treatment of the stump, it may be ligatured or clamped and fixed in the lower angle of the womb, or ligatured and dropped into the abdominal cavity, or turned into the vaginal cavity through an opening in the vaginal fornix. Or the uterine arteries may be ligatured and the stump hollowed out and sutured or left open. Various minor modifications have been employed, such as covering the stump with peritoneal flaps, or stitching it under the abdominal incision, but outside of the peritoneal cavity, etc., etc. Many are now taking out the entire uterus, cervix and all. It is not desirable to discuss here these methods in detail. I will merely state my individual experience as a contribution to the history of the operation. I employ vaginal fixation almost exclusively. Among thirty-four such operations done for fibroids, there have been two deaths and thirty-two recoveries. Time can only tell what method will become the accepted one. The statistics of all methods are improving. Abdominal fixation of the stump is perhaps a trifle the safest, but is so often followed by hernia, and has such a prolonged and disagreeable after-treatment connected with it, that it must be superseded by the first method that will show as good statistics.

---

### LOVE (I. N.) ON RELIEF FROM PAIN IN LABOR.

1. Every pregnant woman throughout the entire period of her pregnancy should have the most careful attention given to her organs of elimination and to proper exercise.

2. Her nerve force throughout the ordeal must be economized, and particularly is this necessary during the last days and hours of her engagement.

3. The well-being of herself and her child is involved in this matter of elimination, exercise and tranquilization.

4. Rest is the great encourager of repair as well as growth; repair to the exhausted force of the woman thus assisting the proper growth of the unborn child.

5. Pain long continued is dangerous, particularly to those not well endowed by nature for the bearing of pain, and as we never think of ignoring the element of shock in our surgical injuries, no more should we ignore it in the parturient state.

6. The rasping, destructive injury to the grosser tissues of the human body are often observed by all surgeons. The more complete the surgical management of the case, the completest possible rest after the injuries favors the completest healing, and yet scars may remain.

6. We should save the nervous systems of the mothers of the world from the rasping, destructive traumatisms produced by labor, and at the same time we should favor rapid repair, never losing sight of the fact that while the healing may be complete, scars yet remain, and in nerve tissues are much more difficult to recover from, and are accompanied by greater interference with proper performance of function than in other more vulgar tissues.

8. Balmy sleep is not only "tired nature's sweet restorer," but also the restorer of burdened, fretted, fagged-out, wounded, wrecked nerve structures.

9. Graves, the great Irish physician, only asked that there be placed upon his epitaph, "He fed fevers." Every thoughtful, warm-blooded, scientific and helpful physician might well ask that there be placed upon his tomb the epitaph, "He gave to his beloved sleep; he saved his patient from pain; not recklessly but intelligently, judiciously, thoughtfully, humanely."—*Jour. Am. Med. Asso., August* 26, 1893.

---

### POST-PARTUM OVARIOTOMY.

Dr. Aust. Lawrence read a paper with this title before the Obstetric Section of the British Medical Association (*Med. Rec.*). He narrated ten cases on which he had operated; in all peritonitis had followed the labor, and the patients were supposed to have puerperal fever, the presence of the cyst not having been suspected during pregnancy. He drew the conclusion that when an ovarian cyst was discovered in the abdomen of a pregnant woman it should be removed as soon as possible, there being many dangers if it were let alone, whereas, on the other hand pregnant women bore abdominal section well. Supposing the cyst were first discovered when the patients actually were in labor, he laid it down that one should endeavor to get the labor over with as little straining as possible, using forceps if admissible, and if symptoms of peritonitis appeared an operation might be performed; otherwise it would be better to wait till the puerperium was passed.

Dr. Pozzi agreed that it was a dangerous thing to have an ovarian cyst in the abdomen of a pregnant woman, the principal accident to which she was liable being the twisting of the pedicle. Sometimes, when the torsion was incomplete there was pain but no rise of temperature. He pointed out that sometimes the swelling in the abdomen was on the opposite side to that on which the cyst grew, and sometimes he had found similar symptoms caused by elongation of the pedicle, the cyst having been lifted up by the pregnant uterus and formed adhesions high up in the abdomen upon which there was tension after the uterus emptied. The symptoms in these cases were chronic and differed from the usually acute symptoms of torsion.

Dr. Byers thought that if a cyst were discovered near the end of term it would be better to wait until after labor before interfering.

Dr. Murphy, the president, had found pregnant women bear all operations well, and thought that in every case of pregnancy it was desirable to operate when a tumor was discovered.—*St. Louis Medical and Surgical Journal.*

---

MAYS (W. H.) ON ANTISEPSIS IN OBSTETRIC PRACTICE.

1. At the onset of labor the external genitals should be cleansed thoroughly with hot water and soap and washed afterwards with 1 to 5,000 bichloride solution.

2. Should an ante-partum vaginal douche be indicated, a hot 1 to 5,000 solution should be gently streamed through the nozzle of a fountain syringe; first into the anterior, then into the posterior pouch of the vagina. Of course this must be done before the rupture of the membrane. Some boiled water should afterwards be injected that there may be no absorption of mercury.

3. Frequent examinations should be avoided. The hands of the physician should be washed in the above solution before and after every examination.

4. Apply napkins wrung out of the solution to the child's head as it emerges from the vulva.

5. See that the external genitals are bathed carefully in the same solution soon after delivery, and at least twice a day afterward. After each bathing and douching an aseptic pad must be applied so as to cover the vulva, reaching to the mons.

6. After the second day commence the daily vaginal douche, consisting of one to two drachms of carbolic acid to the quart of hot water. This may be repeated twice a day if necessary, and should be continued till the lochial discharge stops. In normal labor no attempt should be made to douche the uterine cavity.

7. The strictest precautions as to cleanliness should guard all these manipulations. The nozzle and tube of the syringe should remain in a disinfecting solution when not in use.—*Pacific Medical Journal, August,* 1893.

[We quote the above in order to differ with it. In normal labor, all the above precautions, except No. 3, are entirely superfluous and fail to give any better results than when they are omitted.]

---

### WYHE (W. GILL) ON DYSMENORRHEA AND CHRONIC ENDOMETRITIS.

Undue importance has been attached to retroversion, and other displacements as the direct cause of symptoms. Displacements simply complicate disease, and may give rise to pain, which, however, is due less to the displacement than to other conditions.

When the broad ligaments are thickened, and the tubes and ovraies diseased, pain will be experienced, as the subject assumes the upright position, even when there is no displacement from the downward pressure exerted upon the fixed uterus and diseased ovaries. When the uterus is healthy, displacement causes but little trouble.

If retroversion exist, next determine whether the case be complicated by disease of the endometrium, or by salpingitis.

If disease of the tubes and ovaries be present, the only procedure is their removal. We cannot treat disease of the Fallopian tubes by curetting and draining. We cannot reach the seat of disease except by laparotomy.

If no such complication exist, boroglyceride tampons should be placed twice a week between the menstrual periods—but pessaries have no place in treatment.

If such treatment fail to effect involution and improved circulation, and the introduction of the sound cause bleeding or pain, then douche, curette and drain, making a simple application—never one that will destroy the mucous lining with its deep-seated glands and follicles, leaving a scar that will do more harm than good in after life.

As to the curetting, the sharp instrument only is worthy of use. The blunt copper wire curette may turn up the granulations, but will not remove them, leaving behind a better nidus for sepsis than before the dilatation. When there are large pieces of tissue to be removed, blunt and rounded forceps are to be used.—*Internat. Jour. of Surg., September*, 1893.

---

### ZABALA (L.) ON RESUSCITATION OF THE NEW-BORN.

The instrument suggested consists of two parts, the catheter and the ball. The catheter is metallic and curved in one half whose end fits to the shape of the infant's glottis, with one hole on the outside for the passage of the air. The curve of the catheter is regulated by the convexity of the floor of the mouth. The exterior end is straight and adapted to one opening of the elastic ball. This ball is made of soft rubber, and has two openings, one with a metallic aperture, to receive the outer end of the catheter, and the exterior to allow the entrance of the air. Its capacity is calculated to answer to the inspiratory capacity of a new-born's lungs, thus avoiding the risk of their over-distending.

The infant in Sylvester's position, or better still, in a pail of hot water, as in De Forest's. After cleaning the fauces with my fingers wrapped in a cloth, I enter the mouth again with my bare left index pressing the tongue till I feel the epiglottis; I go as far as filling the cavity of the glottis with the point of the finger. Once there, I hold my catheter and carry it to the larnyx, when I push the end straight into its cavity. I keep it in place with my left hand, while with my right I fix the empty ball to the outside and I next produce a movement of aspiration that answers very well for the purpose of emptying the new-born's trachea and bronchi of all the foreign substances that may have lodged there. I clean, then, the ball and apply it empty again until I believe that the respiratory passages are totally free, and not until then do I force the air into the lungs. The movement of the insufflator ought to be made at the rate of from fifteen to twenty times to the minute and continued with hope of success as long as the beatings of the heart are not totally extinct.—*Loth. Cal. Practitioner, September*, 1893.

